# Supplementary material for: Divergent Pharmacology and Biased Signaling of the Four Melanocortin-4 Receptor Isoforms in Rainbow Trout (Oncorhynchus mykiss)
Source: Biomolecules. 2023 Aug 16;13(8):1248. doi: 10.3390/biom13081248 (PMC10452266; doi:10.3390/biom13081248)
Supplement: Supplementary file 1 [file biomolecules-13-01248-s001.zip › biomolecules-2512447-supplementary.pdf]

Ji *et al.* (2023) Supplementary Figure S1

```
Human POMC            - - - - - - - - - - - M P R S C C S R S G A L L L A L L L - Q A S M E V R G W C L E S S Q C Q D L T T E S N L L E C I R 48
Raibow trout Pomca    - - - - - - - - - - - - - - - M L C - - P A W L L A V A V V - G V V R G V K G Q C W E N P R C H D L S S E N N L L E C I Q 43
Raibow trout Pomcb    M F G T F L Q N Q S V R L N M V C - - A P W L L A V V V V C V C N P G V E G Q C W D S S H C K D L P S E D K I L E C I H 58
Consensus             * * * . : : * . * * : . : * : * * : * . : : * * * * :

                                                                        γ-MSH
Human POMC            A C K P D L S A E T P M F P G N G D E D P L T E N P - - - - R K Y V M G H F R W D R F G R R N S S S S G S S G A G Q K R 104
Raibow trout Pomca    L C R S D L T T K S P I F P V K V H L Q P P S P S D S D S P P L Y L P L S - - - - - - - - - - - - - - - - - - - - - - 80
Raibow trout Pomcb    L F R S G L Q D E S P E P R S A A Q - Q S T E E S - - - - - - - - - - - - - - - - - - - - - - - - - - - - - - - - - - 82
Consensus             : . * : : * . * .

                                                                                            ACTH
                                                                        α-MSH
Human POMC            E D V S A G E D C G P L P E G G P E P R S D G A K P G P R E G K R S Y S M E H F R W G K P V G K K R R P V K V Y P N G A 164
Raibow trout Pomca    - - - - - - - - - - L L S P S S P L Y P T E Q Q N S V S P Q A K R S Y S M E H F R W G K P V G R K R R P V K V Y T N G V 130
Raibow trout Pomcb    - - L S L G I L L A A L T S G E - - R A L D A D P E P H S D K R H S Y S M E H F R W G K P I G H K R R P I K V Y A S S L 138
Consensus             * . : : : : * * * * * * * * * * * * * : * : * * * * : * * * . .

Human POMC            E D - - E S A E A F P L E F K R E L T G Q R L R E G D G P D G P A D D G A G A Q A D - - L E H S L L V A A E K K D E G P 220
Raibow trout Pomca    E E - - E S S E A F P S E M R R E L G T D D A V Y P S L E A G T A E G G E A E - - G - - M - - E G V F S L Q E K K D G S 182
Raibow trout Pomcb    E G G D S S E G T F P L Q A R R Q L S S W E D E M V - - - G A L G N Q G A K A Q T K V V P R T L T V T G L Q D K K D G S 195
Consensus             * . * : * * : : * : * . . : * : . : . * . : *

                            β-MSH
Human POMC            Y R M E H F R W G S P P K D K R Y G G F M T S - - E K S Q T P L V T L F K N A I I K N A Y K K G E - - - - - - - - - - - 267
Raibow trout Pomca    Y K M N H F R W S G P P A S K R Y G G F M K S W D E R S Q K P L L T L F K N V I I K D G Q Q K R E Q W G R E E G E E K R 242
Raibow trout Pomcb    Y R M G H F R W G S P T A I K R Y G G F M K P Y T Q Q S H K P L I T L L K H V T L K N E Q - - - - - - - - - - - - - - - 240
Consensus             * : * * * * * . . * * * * * * * * . : : * : . * * : * * : * : . : * :

Human POMC            - - - - - - - - - - - -                                                                                                  267
Raibow trout Pomca    A L G E R K Y H F Q G                                                                                                    253
Raibow trout Pomcb    - - - - - - - - - - - -                                                                                                  240
Consensus
```

Ji *et al.* (2023) Supplementary Figure S2A

1	ATG	AAT	GCC	ACG	CAC	CAG	CAT	CAC	CAT	GGA	TCA	TTT	CAC	CTA	TGG	AAC	CAC	AGC	TCT	GGA	60
1	M	N	A	T	H	Q	H	H	H	G	S	F	H	L	W	N	H	S	S	G	20
61	GCT	CCG	CCT	CTA	AGC	CAT	CAA	CAG	CAC	CAA	GCC	GGA	GCG	GAG	AGA	CAC	CAT	GGT	TCG	TCT	120
21	A	P	P	L	S	H	Q	Q	H	Q	A	G	A	E	R	H	H	G	S	S	40
121	GGG	TGT	TAC	GAG	CAG	CTG	CTA	ATC	TCC	ACC	GAG	GTC	TTC	CTC	ACG	CTG	GGC	ATC	GTC	AGC	180
40	G	C	Y	E	Q	L	L	I	S	T	E	V	F	L	T	L	G	I	V	S	60
181	CTG	CTG	GAG	AAC	ATC	CTG	GTC	ATC	GCC	GCC	ATC	ATT	AAG	AAC	AAG	AAT	CTG	CAC	TCT	CCC	240
61	L	L	E	N	I	L	V	I	A	A	I	I	K	N	K	N	L	H	S	P	80
241	ATG	TAC	TTC	TTC	ATC	TGT	TCC	CTG	GCC	ATG	GCC	GAC	ATG	CTG	GTC	AGC	GTC	TCC	AAT	GCC	300
81	M	Y	F	F	I	C	S	L	A	M	A	D	M	L	V	S	V	S	N	A	100
301	ACC	GAG	ACC	ATC	GTT	ATG	GCA	ATG	ATC	ACC	GAC	GGA	AAT	CTG	GGG	ATT	GGT	GGC	GGC	GTG	360
101	T	E	T	I	V	M	A	M	I	T	D	G	N	L	G	I	G	G	G	V	120
361	ATC	AAG	AGC	ATG	GAC	AAC	GTG	TTT	GAC	TCC	ATG	ATC	TGT	AGT	TCC	CTG	CTG	GCG	TCT	ATC	420
121	I	K	S	M	D	N	V	F	D	S	M	I	C	S	S	L	L	A	S	I	140
421	TGG	AGC	CTG	CTG	GCC	ATC	GCC	GTG	GAC	CGT	TAC	GTG	ACC	ATC	TTC	TAC	GCG	CTG	CGC	TAC	480
141	W	S	L	L	A	I	A	V	D	R	Y	V	T	I	F	Y	A	L	R	Y	160
481	CAC	AAC	ATC	ATG	ACC	ACG	CGC	CGG	GCC	GCC	GCC	ATC	ATC	ACC	AGC	ATC	TGG	ACC	TTC	TGC	540
161	H	N	I	M	T	T	R	R	A	A	A	I	I	T	S	I	W	T	F	C	180
541	ACC	GTG	TCG	GGC	GTC	CTC	TTC	ATC	GTC	TAC	TCG	GAG	AGC	ACT	ACC	GTC	CTC	ATC	TGC	CTC	600
181	T	V	S	G	V	L	F	I	V	Y	S	E	S	T	T	V	L	I	C	L	200
601	ATC	ATC	ATG	TTC	TTC	AGC	ATG	CTG	GTG	CTC	ATG	GCC	TCG	CTG	TAC	GTC	CAC	ATG	TTC	ATG	660
201	I	I	M	F	F	S	M	L	V	L	M	A	S	L	Y	V	H	M	F	M	220
661	CTG	GCA	CGT	CTG	CAC	ATG	AAG	AGG	ATC	GCC	GTT	CTG	CCG	GGA	AAC	GGC	CCC	ATC	TGG	CAG	720
221	L	A	R	L	H	M	K	R	I	A	V	L	P	G	N	G	P	I	W	Q	240
721	GCA	GCC	AAC	ATG	AAG	GGG	GCC	ATC	ACC	CTC	ACC	ATT	CTC	TTG	GGG	GTG	TTC	ATA	GTG	TGC	780
241	A	A	N	M	K	G	A	I	T	L	T	I	L	L	G	V	F	I	V	C	260
781	TGG	GCT	CCT	TTC	TTC	CTC	CAC	CTC	ATC	CTC	ATG	ATC	TCC	TGC	CCT	AGG	AAC	CCC	TAC	TGT	840
261	W	A	P	F	F	L	H	L	I	L	M	I	S	C	P	R	N	P	Y	C	280
841	GTG	TGC	TTC	ATG	ACC	CAC	TTT	AAC	ATG	TAC	CTC	ATC	CTC	ATC	ATG	TGT	AAC	TCT	GTC	ATT	900
281	V	C	F	M	T	H	F	N	M	Y	L	I	L	I	M	C	N	S	V	I	300
901	GAC	CCG	CTG	ATC	TAC	GCC	TTC	AGG	AGC	CAG	GAG	ATG	AGG	AAA	ACC	TTC	AAG	GAG	ATC	TTC	960
301	D	P	L	I	Y	A	F	R	S	Q	E	M	R	K	T	F	K	E	I	F	320
961	TGC	TGT	TGG	TAT	GGT	CTC	GCC	TCT	GTG	TGT	TTC	TCT	GTG	TGA							
321	C	C	W	Y	G	L	A	S	V	C	F	S	V	*							

Ji *et al.* (2023) Supplementary Figure S2B

1 ATG AAT GCC ACG CAC CAG CAT CAC CAT GGA TCA TTC CAC CTA CGG AAC CAC AGC TCT GGA 60
1 M N A T H Q H H H G S F H L R N H S S G 20
61 GCT CTA CCT TTC AGC AAT CAG CAG CCC CAG GCT ATG GCA GAG AGA TTC CGT GGT TTG CCT 120
21 A L P F S N Q Q P Q A M A E R F R G L P 40
121 GGG TGT TAT GAG CAG CTG CTC ATC TCC ACT GAG GTC TTC CTC ACG CTG GGC ATC GTC AGC 180
40 G C Y E Q L L I S T E V F L T L G I V S 60
181 CTG CTG GAG AAC ATC CTA GTT ATT GCC GCC ATC ATC AAG AAC AAG AAT CTT CAC TCT CCC 240
61 L L E N I L V I A A I I K N K N L H S P 80
241 ATG TAC TTC TTC ATC TGT TCC CTG GCC GTG GCT GAC ATG CTG GTC AGC GTC TCC AAC GCC 300
81 M Y F F I C S L A V A D M L V S V S N A 100
301 ACT GAG ACC ATT GTC ATG GCG ATG ATC ACT GAT GGC AAC TTG GGG ATC GGC GGT GGC ATG 360
101 T E T I V M A M I T D G N L G I G G G M 120
361 ATC AAG AGC ATG GAC AAC GTG TTT GAC TCC ATG ATC TGT AGT TCC CTG CTG GCG TCT ATC 420
121 I K S M D N V F D S M I C S S L L A S I 140
421 TGG AGC CTG CTG GCC ATC GCT GTT GAC CGT TAC GTG ACT ATC TTC TAT GCA CTA CGC TAC 480
141 W S L L A I A V D R Y V T I F Y A L R Y 160
481 CAC AAC ATT ATG ACC GTG CGC CGG GCT GCC ACC ATC ATC ACC AGC ATC TGG ACC TTC TGC 540
161 H N I M T V R R A A T I I T S I W T F C 180
541 ACC GTG TCG GGC GTC CTC TTC ATC GTC TAC TCG GAG AGC ACT ACC GTC CTC ATC TGC CTC 600
181 T V S G V L F I V Y S E S T T V L I C L 200
601 ATC ATC ATG TTC TTC AGC ATG CTG GTG CTC ATG GCC TCG CTG TAC GTC CAC ATG TTC ATG 660
201 I I M F F S M L V L M A S L Y V H M F M 220
661 CTG GCG CGT CTG CAC ATG AAG AGG ATC GCC GCT CTG CCG GGC AAC GGC CCC ATC TGG CAG 720
221 L A R L H M K R I A A L P G N G P I W Q 240
721 GCA GCC AAC ATG AAG GGG GCC ATC ACC CTC ACC ATC CTC CTG GGG GTG TTC ATA GTG TGC 780
241 A A N M K G A I T L T I L L G V F I V C 260
781 TGG GCG CCC TTC TTC CTC CAT CTC ATC CTC ATG ATC TCC TGC CCC AGG AAC CCC TAC TGT 840
261 W A P F F L H L I L M I S C P R N P Y C 280
841 ATG TGC TTC ATG TCT CAC TTC AAC ATG TAC CTC ATT CTC ATC ATG TGT AAC TCT GTC ATC 900
281 M C F M S H F N M Y L I L I M C N S V I 300
901 GAC CCG CTA ATC TAC GCC TTC AGG AGC CAG GAG ATG AGG AAG ACC TTC AAG GAG ATA TTC 960
301 D P L I Y A F R S Q E M R K T F K E I F 320
961 TGC TGC TGG TAT GAT ATT GCC TCT CTG TGT GTC TCT GTG TGA
321 C C W Y D I A S L C V S V *

1	ATG	ATG	AAT	TCC	ACA	GAC	CAC	CAA	GGG	TTG	ATC	TCT	GTG	GGC	TAT	ACC	AGG	AAC	CTC	AGC	60
1	M	M	N	S	T	D	H	Q	G	L	I	S	V	G	Y	T	R	N	L	S	20
61	ACT	GCT	GGG	ACT	CTG	GGA	ACC	CTC	AAC	AAA	GAC	TCA	GAG	GGC	GTT	GGT	ATC	AAG	GAC	TCC	120
21	T	A	G	T	L	G	T	L	N	K	D	S	E	G	V	G	I	K	D	S	40
121	TCA	ACA	GGA	TGT	TAC	GAG	CAG	CTC	CTC	ATC	TCT	ACC	GAG	GTC	TTT	CTC	ACA	CTG	GGG	ATA	180
40	S	T	G	C	Y	E	Q	L	L	I	S	T	E	V	F	L	T	L	G	I	60
181	GTC	AGT	TTA	TTA	GAG	AAC	ATC	CTG	GTG	ATT	GCT	GCC	ATC	ATC	AAG	AAT	AAG	AAT	CTT	CAC	240
61	V	S	L	L	E	N	I	L	V	I	A	A	I	I	K	N	K	N	L	H	80
241	TCT	CCC	ATG	TAC	TTA	TTC	ATC	TGT	TCT	TTG	GCT	GTG	GCA	GAC	ATG	CTG	GTC	AGC	GTC	TCC	300
81	S	P	M	Y	L	F	I	C	S	L	A	V	A	D	M	L	V	S	V	S	100
301	AAC	GCC	TCC	GAG	ACC	ATC	GTC	ATC	GCC	CTG	ATC	AAC	GGC	GGC	AAC	TTG	ACC	ATC	TCC	GGG	360
101	N	A	S	E	T	I	V	I	A	L	I	N	G	G	N	L	T	I	S	G	120
361	TCG	CTC	ATA	AAG	AGC	ATG	GAC	AAC	GTG	TTC	GAC	TCC	ATG	ATC	TGT	AGC	TCA	CTG	CTG	GCG	420
121	S	L	I	K	S	M	D	N	V	F	D	S	M	I	C	S	S	L	L	A	140
421	TCA	ATC	TGT	AGT	CTC	TTG	GCC	ATC	GCC	ATA	GAC	CGC	TAC	ATC	ACC	ATA	TTC	TAC	GCG	CTG	480
141	S	I	C	S	L	L	A	I	A	I	D	R	Y	I	T	I	F	Y	A	L	160
481	CGC	TAC	CAT	AAC	ATT	GTG	ACG	GTA	AAG	CGA	GCG	ATG	GCG	GTA	ATC	GCG	TGC	ATC	TGG	TCG	540
161	R	Y	H	N	I	V	T	V	K	R	A	M	A	V	I	A	C	I	W	S	180
541	TGT	TGT	GTG	GCA	TCG	GGC	GTG	CTC	TTC	ATT	ATC	TAC	TCT	GAG	AGC	ACC	ACG	GTC	CTC	ATC	600
181	C	C	V	A	S	G	V	L	F	I	I	Y	S	E	S	T	T	V	L	I	200
601	TGC	CTC	ATC	ACC	ATG	TTC	TTC	ACC	ATG	CTG	GCG	CTC	ATG	GCC	TCT	CTC	TAC	GTC	CAC	ATG	660
201	C	L	I	T	M	F	F	T	M	L	A	L	M	A	S	L	Y	V	H	M	220
661	TTC	ATG	CTG	GCC	CGC	CTG	CAC	ATA	AAG	AGG	ATT	GCC	GTT	CTG	CCC	GGG	AAC	GTT	CCC	ATC	720
221	F	M	L	A	R	L	H	I	K	R	I	A	V	L	P	G	N	V	P	I	240
721	CGC	CAG	CGT	GCC	AAC	ATG	AAG	GGC	GCC	ATC	ACC	CTC	ACC	ATC	CTC	CTG	GGT	GTG	TTC	GTA	780
241	R	Q	R	A	N	M	K	G	A	I	T	L	T	I	L	L	G	V	F	V	260
781	GTG	TGC	TGG	GCT	CCC	TTT	TTC	CTC	CAC	CTC	ATC	CTC	ATG	ATA	TCA	TGC	CCC	AGG	AAC	CCC	840
261	V	C	W	A	P	F	F	L	H	L	I	L	M	I	S	C	P	R	N	P	280
841	TAC	TGT	GCC	TGC	TTC	ATG	TCA	CAC	TTC	AAC	ATG	TAC	CTC	ATC	CTC	ATC	ATG	TGT	AAC	TCT	900
281	Y	C	A	C	F	M	S	H	F	N	M	Y	L	I	L	I	M	C	N	S	300
901	GTC	ATC	GAC	CCA	CTG	ATC	TAC	GCC	TTC	AGG	AGC	CAA	GAG	ATG	AGG	AAG	ACC	TTT	AAG	GAA	960
301	V	I	D	P	L	I	Y	A	F	R	S	Q	E	M	R	K	T	F	K	E	320
961	ATC	TTC	TGC	TGG	TAC	AGT	CTG	CCA	AAC	CTG	TGT	GTG	TGC	GAG	CTG	CCA	GGG	AAA	TAT	TGA	1020
321	I	F	C	W	Y	S	L	P	N	L	C	V	C	E	L	P	G	K	Y	*	339

Ji *et al.* (2023) Supplementary Figure S2D

1 ATG AAT GCC ACA GAA TAC CAG GGG TCG ATC TCT GTG AGC TAT AAC AGG AAC TCC AGC ACT 60

1 M N A T E Y Q G S I S V S Y N R N S S T 20

61 GCT GGG ACT CTG GTA TCC GTC AAC AAA GAC TCA GAG GGC ATT GGT CTC AAT GAC TCT TCG 120

21 A G T L V S V N K D S E G I G L N D S S 40

121 ACA GGA TGT TAC GAC CAG CTC CTC ATC TCC ACC GAG GTC TTC CTC ACA CTG GGG ATT GTC 180

40 T G C Y D Q L L I S T E V F L T L G I V 60

181 AGT TTA TTA GAG AAC ATC CTG GTG ATT GCC GCT ATC ATC AAG AAC AAG AAC CTC CAC TCT 240

61 S L L E N I L V I A A I I K N K N L H S 80

241 CCC ATG TAC TTC TTC ATC TGT TCT CTG GCT GTG GCC GAC ATG CTG GTC AGC GTC TCC AAT 300

81 P M Y F F I C S L A V A D M L V S V S N 100

301 GCC TCC GAG ACG ATC GTC ATA GCC CTG ATC AAC AGC GGC AGC CTG AGC ATC TCT GGG TCA 360

101 A S E T I V I A L I N S G S L S I S G S 120

361 CTG ATA AAA AGC ATG GAC AAT GTG TTC GAC TCC ATG ATC TGT AGC TCA CTG CTG GCA TCA 420

121 L I K S M D N V F D S M I C S S L L A S 140

421 ATC TGT AGT CTC TTG GCA ATC GCC ATA GAC CGC TAC ATC ACC ATA TTC TAT GCG CTG CAC 480

141 I C S L L A I A I D R Y I T I F Y A L H 160

481 TAC CAT AAC ATT GTG ACG GTA AAA AGA GCG CTG GCA GTG ATC TCC CTC ATA TGG TTG TGT 540

161 Y H N I V T V K R A L A V I S L I W L C 180

541 TGC GTG GTG TCG GGC GTG CTC TTC ATC ATC TAC TCT GAG AGC ACC ACT GTC CTC ATC TGC 600

181 C V V S G V L F I I Y S E S T T V L I C 200

601 CTT ATC ACC ATG TTC TTC ACC ATG CTG GCC CTC ATG GCC TCG CTC TAC ATC CAC ATG TTC 660

201 L I T M F F T M L A L M A S L Y I H M F 220

661 CTC CTA GCC CGG CTA CAC ATG AAG AGG ATC GCC ATG CTG CCT GGG AAC GCG CCC ATC CGC 720

221 L L A R L H M K R I A M L P G N A P I R 240

721 CAG CAT GCC AAC ATG AAG GGT GCC ATC ACC CTC ACC ATC CTC CTA GGT GTG TTC GTA GTG 780

241 Q H A N M K G A I T L T I L L G V F V V 260

781 TGC TGG GCT CCC TTC TTC CTC CAC CTC ATC CTC ATT ATC TCC TGC CCT AGG AAT CCC TAC 840

261 C W A P F F L H L I L I I S C P R N P Y 280

841 TGT GCC TGC TTC ATG TCG CAC TTC AAC ATG TAC CTC ATC CTC ATC ATG TGT AAC TCG GTC 900

281 C A C F M S H F N M Y L I L I M C N S V 300

901 ATC GAC CCA CTC ATC TAC GCA CTC CGC AGC CAG GAG ATG AGG AAG ACC TTC AAG GAG ATC 960

301 I D P L I Y A L R S Q E M R K T F K E I 320

961 TTC TGC TGG TAC AGC TTG CCA AAC CTG TGT GTG TGT GAG CTC CCT GGG AAA TAG

321 F C W Y S L P N L C V C E L P G K *
